# Benefits of GP care in outreach settings for people experiencing homelessness: a qualitative study

**DOI:** 10.3399/BJGP.2020.0749

**Published:** 2021-06-02

**Authors:** Victoria Hirst, Fiona Cuthill

**Affiliations:** Edinburgh Access Practice, Edinburgh.; School of Health in Social Science, University of Edinburgh, Edinburgh.

**Keywords:** community outreach, homelessness, patient engagement, primary care

## Abstract

**Background:**

Although people experiencing homelessness (PEH) have the worst health outcomes in society, they have a low uptake of primary care services. GP outreach has developed as a way of increasing their access into primary care but little is known about the experience of patients receiving care in this way.

**Aim:**

To explore PEHs’ experiences of GP care in community outreach settings in UK; and to seek staff/volunteers’ views on the strengths and weaknesses of GP community outreach services.

**Design and setting:**

A multi-method qualitative study with PEH and staff/volunteers working in three different community outreach settings in the UK.

**Method:**

Individual semi-structured interviews were carried out with 22 PEH and two focus groups with key staff/volunteers. Data were analysed thematically using framework analysis.

**Results:**

GP outreach services better enabled PEH to access medical care and staff/volunteers valued GP support to promote, and facilitate access to, healthcare services. In particular, the findings illuminate the high value that PEH placed on the organisational environment of the GP outreach service. Valued aspects of GP outreach were identified as comfortable, safe, and engendering a sense of belonging; convenient, opportunistic, and a one-stop shop; and being heard, having more time, and breaking down barriers.

**Conclusion:**

The organisational environment is important in enabling PEH to engage with GP services. The physical and organisational environment of the outreach settings were the most important factors; they created a space where professional barriers between the GP and patients were flattened, so facilitating a therapeutic relationship.

## INTRODUCTION

Homelessness has been recognised for more than a decade as a healthcare issue.^1^ It is a ‘late marker’ of severe and complex disadvantage,^2^ and the result of enduring health and social inequities.^3^ A 2014 health audit of over 2500 people experiencing homelessness (PEH) in England found a higher prevalence of physical, mental, and substance misuse issues in the homeless population compared with the general population.^4^ Standardised mortality rates for excluded populations, including PEH, are 7.9 times higher for men and 11.9 times higher for women.^5^ PEH are at increased risk of non-communicable diseases,^5^ infectious disease,^6^^,^^7^ and multimorbidity.^8^

Nonetheless, PEH often experience substantial barriers to accessing health care and report feeling excluded from health services, including mainstream substance misuse and mental health services.^9^^,^^10^ Barriers to accessing mainstream primary care services include inflexibility of the healthcare system,^11^ negative staff attitudes,^12^ difficulties with GP registration following hospital or prison discharge,^8^ and a lack of specialist primary care centres for PEH.^9^ While continuity of care from secondary to primary care is recognised in the UK as crucial for PEH,^13^ the reality is often discharge to the streets, resulting in fragmentation and delay of treatment.

Nearly one in three deaths of PEH are due to causes amenable to effective healthcare interventions,^5^ and GPs play an important role in homelessness prevention.^6^ The first data linkage study in Scotland between health and homelessness services found that, in the months immediately preceding a first episode of homelessness, visits to mainstream GP services increased.^14^ Nonetheless, it seems that preventive opportunities are often missed. While specialist primary care centres for PEH exist in many areas,^15^ increasing access to healthcare services for those who engage,^9^ many PEH do not access primary care services at all, exacerbating already poor health outcomes and increasing the risk of premature death.^8^ This is particularly concerning given the steep rise in the numbers of PEH in the UK since 2010,^16^ particularly in England, and the projected recession following the COVID-19 global pandemic, which is expected to trigger a wave of people who are newly-homeless as unemployment increases and people are unable to secure affordable housing.^17^

In response to these challenges, GP outreach services to hostels, the streets, and day centres have been developed to increase access to primary care services for PEH.^15^ As these services have developed in an ad hoc manner, they vary greatly in scope of provision across different areas, and little is known about the experiences of PEH when GP care is delivered in this way. This study therefore sought to more fully understand what PEH and frontline staff and volunteers working in community settings value about GP outreach services, so that primary care services can be improved to meet the needs of PEH and reduce health inequalities.

**Table:** How this fits in

Although people experiencing homelessness (PEH) have the worst health outcomes in society, they have a low uptake of primary care services. GP outreach has developed as a way of increasing their access to primary care but little is known about the experience of patients receiving care in this way. By exploring the experiences of PEH and staff/volunteers working in community outreach settings, this study uncovers the reasons why PEH engage with GPs in a community outreach setting but not a specialist or mainstream GP service. Clearly, the physical space and organisational environment of outreach settings are important factors. These findings can help to inform GPs caring for PEH to build an environment which supports the development of stronger doctor–patient relationships within the confines of their current system.

The aims of this study were to explore PEHs’ experiences of GP care in community outreach settings in England and Scotland; and to seek staff/volunteers’ views on the strengths and weaknesses of the GP community outreach services. The research questions the study sought to answer were what are the enablers and barriers to using GP outreach services as experienced by PEH and what do staff/volunteers see as the advantages and disadvantages of a GP service in an outreach setting?

## METHOD

This study used an interpretivist approach to explore the experiences of PEH when using GP outreach services. Interpretivism is based on an understanding of human experience that relies on the reflection and interpretation of lived experience to uncover how participants make sense of their individual and social worlds. Interpretivism seeks to understand these experiences within the context of people’s lives, as opposed to methodologies that seek to create an objective statement of the experience itself.^18^ Therefore, consistent with interpretivism, this study sought to recognise meaning from the experiences of homeless people and staff/volunteers of GP outreach services from their point of view.

Themes and ideas are derived from the analysis of the data and hence the interpretation is grounded in, and supported by, the data.

### Design

A multiple-method qualitative study was conducted with two phases, which took place between May and September 2017. Phase one involved semi-structured face-to-face interviews with 22 PEH. Phase two involved conducting two focus groups with key staff/volunteers. Key staff included those working in three different community settings in the UK: one in Scotland (setting 1) and two in Northern England (settings 2 and 3). Details of the study settings are outlined in Table 1.

**Table 1.** Details of study settings

**Setting**	**Type**	**Services**	**Hours**	**GP outreach^a^**	**Appointments system**
1	Drop-in day-centre	Shower facilities, washing machines, support work, internet access	7am to 10pm Every day	1 regular day/week 8am to 12 noon	PEH seen in order of arrival. Patients continued with usual activities at the setting while waiting, such as using computers, chatting in lounge area, and using showers.
2	Food drop-in	Breakfast and hot lunch, clothing bank, literacy teaching, housing/legal advice, hairdresser	9am to 1pm 1 day/week	1 regular day/week 9am to 12 noon, GP present at every drop-in session	PEH seen in order of arrival. Patients continued with usual activities at the setting while waiting, such as seeing advisers, getting lunch, using clothing bank.
3	Food drop-in	Drink and hot meal, clothing bank	9am to 11am 6pm to 8pm Twice weekly	GP present at every drop-in session	PEH seen in order of arrival. Patients continued with usual activities at the setting while waiting, such as having hot meal or using the clothing bank.

*PEH = People experiencing homelessness.*

a *All GPs who worked as part of the outreach service were based at and supported by the local specialist primary care centre for PEH. In all settings, GPs had use of a private non-clinical space and could chat with PEH anywhere in the setting where appropriate.*

### Sampling

Convenience sampling was used to invite people who had used the GP outreach service, and were currently, or recently, homeless, to explore their experiences. Sampling across three different settings ensured a range of experiences were reflected in the interview data. Key staff/volunteers were invited to participate in two focus groups (settings 1 and 2) to explore their views on the GP outreach service. Study information posters were displayed in all three settings and community setting staff/volunteers explained the study to participants and invited them to participate. Before data collection, written consent was obtained and, where written consent was not possible because of poor literacy, the patient’s oral consent was obtained and recorded digitally. All participants in the interviews were assured of anonymity and confidentiality, and pseudonyms are used throughout. In the focus groups, participants were reminded to maintain confidentiality within the group in accordance with good ethical practice. Interviews and focus groups were conducted in a private room in the community settings, and the PEH were given £20 supermarket shopping vouchers in appreciation of their time.

### Participants

Of the 22 PEH interviewed (10 in the Scottish setting, 12 in the English settings), 15 were male and seven female, and 15 were born in the UK while five were born abroad (three EU nationals and two refused asylum seekers); this information was not given by the two other participants. The age of participants ranged from 19 to 60 years old. Participants who identified as currently homeless were either sleeping on the streets, or living in boarding houses or hostels. Those who had recently been homeless were now renting somewhere of their own.

While the researchers did not explicitly ask about health needs, homeless participants disclosed a range of physical and mental health issues, including harmful substance use, infectious diseases, coronary artery disease and diabetes, and depression, anxiety, and bipolar disorder. All of the participants in the interviews had been to see the GP in the outreach setting in the last year, varying from one visit to regular weekly visits.

Of the two focus groups conducted with staff/volunteers, one took place in Scotland (*n* = 4 staff) and the other in setting 2 (*n* = 7 volunteers). Both focus groups explored the reasons staff/volunteers thought that PEH attended to see a GP in this community setting.

### Data analysis

The interviews and focus groups were recorded and transcribed verbatim. Individually identifiable data were removed and data were analysed thematically using framework analysis, involving five stages of analysis: familiarisation with the data; construction of an initial thematic framework; sorting and indexing the data; creating a framework matrix; and comparing and interpreting the data.^18^

Framework analysis is not attached to any particular theoretical or epistemological position and provides a structured and rigorous process of data management, while simultaneously offering a flexibility that can be applied to a variety of research interests and theoretical positions to facilitate the analysis of patterns or themes within the data.^18^ A distinctive feature of framework analysis is that it forms the basis of a series of thematic matrices where data can then be compared and contrasted, which allowed the researchers to move back and forth between different levels of abstraction without losing sight of the raw data. Data were analysed by both researchers, individually and then together, to ensure that the framework was agreed and consistent across the whole dataset. Data were organised using NVivo (version 10) software.

## RESULTS

This study found that GP outreach services better enabled PEH to access medical care, with additional benefits in supporting staff/volunteers in community settings to give better health advice. This was consistent across all three settings, which is interesting given the differences between the day centre and the food bank settings. The reasons that PEH engaged with healthcare services in outreach settings, but would not attend specialist primary care centres for PEH or mainstream GP practices, were reported as being because of the positive physical, social, and organisational environment of the outreach settings. Compared with GP practices, outreach settings were reported as being more comfortable, were perceived as safer, and engendered a sense of belonging; they were convenient as they brought services together as a ‘one-stop shop’; and patients felt that they were listened to more as the outreach environment was more relaxed and the GPs had more time.

While the staff/volunteers noted a lack of clinical tests and treatment facilities in the community outreach settings, they recognised them as being additional to the specialist primary care centres for PEH, rather than a replacement service. Staff/volunteers said that they valued the support GPs gave them to better meet the ongoing healthcare needs of their clients and to facilitate access to health services.

### A comfortable, safe environment and a sense of belonging: *‘It’s just like one big family’*

Participants attended to see GPs in the outreach settings because they reported that they felt comfortable, safe, and had a sense of belonging. This was contrasted with mainstream GP services or specialist primary care centres for PEH, which were often seen as places where the waiting rooms were restrictive, stressful, and heightened tensions. Staff/volunteers noted that in the community outreach settings, the participants did not have to sit silently waiting for the GP, which made them feel frustrated, often exacerbating underlying mental health problems. Instead, they engaged with support workers and/or volunteers and had other meaningful activity while waiting to see the GP, such as washing clothes or collecting food.

#### Comfortable

The participants reported feeling more relaxed at the outreach setting than at specialist primary care centres for PEH. The environment felt less formal and more familiar — some participants even described it as their ‘own environment’. The staff/volunteers at the outreach settings noted that the PEH attending appeared relaxed and felt that this enhanced the doctor–patient relationship. The staff/volunteers were an integral part of creating this friendly, welcoming environment:

*‘They go into the doctors and sit in a formal room … and they find it hard to say what is wrong with them in that sort of environment. I think when they come in this environment they are already relaxed and calm, and probably more truthful with the doctor.’*

(Volunteer [V], England)

Participants all reported the difference it made to have someone to talk to in the outreach setting, whether that was with staff/volunteers or other guests, and this made the atmosphere much more conducive to waiting for the GP:

*‘It’s ok going to the GP surgery but, when you go to the GP surgery, you are just sat there, you know, and there is nobody talking to you. Here it is different, you are free to talk, you know, like people to talk to while you are waiting. That’s a really good thing, it calms you down as well.’*

(PEH participant [PEHP], aged 28 years, Scotland)

#### Safe

Many participants in Setting 1 reported difficulties in specialist primary care centres for PEH, and in GP waiting rooms with meeting people who they described as *‘drug dealers’* and from a *‘previous life’* so they avoided the GP surgeries. Several participants reported feeling unsafe, threatened or intimidated, as specialist primary care centres for PEH and GP waiting rooms did not have any support workers to mediate the space:

*‘I hate going to the* [specialist primary care centre for PEH] *surgery as you’re trying to get clean and there’s dealers there and aggro and it’s nae right, you know. It’s nae a good place to be and you can get threatened and fights and everything.’*

(PEHP, 34 years, Scotland)

In addition, participants felt that they were treated with respect in the outreach settings, whereas many PEH felt that there was a lack of respect by staff at mainstream practices.

#### A sense of belonging

A sense of belonging that the support workers/volunteers engendered was clearly valued in the outreach settings: participants felt like they were ‘part of one big family’. Of particular value was being known by name; having a relationship with the support workers/volunteers; and a feeling of equity. In the GP service, many participants said that they felt ignored, just waiting around for the GP, but in the outreach setting they were noticed and welcomed:

*‘When I walk through the door I get greeted, you know, normally by name, and they (the volunteers) ask me how I am doing and stand and chat, and they will ask me if I need the doctor or anything else that is here that day.’*

(PEHP, 60 years, England)

Relationships with staff/volunteers appeared to be important in facilitating engagement with the GP and outreach settings. Staff/volunteers talked about the priority they placed on getting to know people who used their services:

*‘We try to get to know people, to speak to them and, if they open up, to help them to access what they need. Some never go to the GP but really need to, so we try to help them. It’s all about building up trust so people open up to you.’*

(Staff [S], Scotland)

Participants reported that they were more likely to see a GP if encouraged by a member of staff who they trusted.

### Convenient, opportunistic, and a one-stop shop: *‘It’s a bonus thought here’*

Health needs were often a low priority for PEH, even though some of the participants had severe health needs. Participants prioritised washing clothes, collecting food, and having a cup of tea/food. Health care was seen as an opportunistic event that was good to have if available, but not something to especially seek out:

*‘I come here for a shower and breakfast, not to see a doctor, but if there’s one here, then I might as well and they’ve started me on some pills now … I never really went to the doctor before.’*

(PEHP, 32 years, Scotland)

As a result, the GP outreach settings were seen a convenient places to see the GP and health was described as *‘a bonus thought here’* (HP, 45 years, Scotland). It was described as a ‘one-stop shop’ for health care, food, washing, and other needs.

#### Convenience

Participants said that they found the GP appointment system in mainstream services particularly difficult to negotiate, especially for those who were sleeping on the streets. Many were awake late into the night and slept for much of the morning when the streets were safer, so they could access the community outreach settings as a drop-in whenever it was convenient for them:

*‘Sometimes it is difficult to get up at 9 o’clock because of whatever circumstances you are in, so it is easier just to see the doctor here* [outreach setting] *… there is always a meeting place where you can meet them where it is actually convenient for you, you know what I mean.’*

(PEHP, 36 years, Scotland)

Staff/volunteers noted the importance of meaningful activity available at the outreach setting:

*‘It’s all a bit formal isn’t it* [at the doctors surgery], *and a bit stuffy. They feel dirty, they feel unclean … they don’t fit. Here they can have a cup of tea, have their breakfast, see the doctor, have some lunch, they can chat …’*

(V, England)

#### Opportunistic and a ‘one-stop shop’

As health care was not a priority for many PEH, participants highlighted the convenience of seeing a GP at the outreach settings and were actively approached by the support workers/volunteers or the GPs themselves to ask if they would like to see a GP:

*‘There are several people here who have never been to see the GP and just won’t go. I don’t know why — maybe a trust thing or bad experience in the past. If we can get to know people and build up trust, they will see the GP here and then go up to the surgery.’*

(S, Scotland)

*‘I stumbled across it [the GP outreach service] at first. I was just told it was a food place and they clothe you and give you sleeping bags and what not because I was on the streets when I first came, and then I got told there was a doctor here and they came round with a form and I put my name down.’*

(PEHP, 40 years, England)

Staff agreed that it was convenient for the PEH to see a GP at the outreach settings but also cautioned that this ‘open access’ system would not work if their numbers increased.

### Being heard, having more time, and breaking down barriers: *‘Less of a white coat syndrome’*

Staff/volunteers and patients noted that the relaxed, non-clinical atmosphere in the outreach settings contributed to good communication between GPs and patients, resulting in stronger relationships and the breaking down of barriers:

*‘When the doctor started coming here, it changed their attitude towards GPs … they felt safe in this environment to talk to them.’*

(V, England)

#### Being heard and having more time

Participants reported feeling grateful that the GP had come to see them in ‘their environment’. At the outreach setting, the GP was afforded more flexibility in their working. This allowed them more time to tailor the sessions to the particular needs of the patients on the day. This resulted in participants feeling less rushed by the GP and that their needs were being individually met:

*‘A homeless guy actually said “Darren, there is a place up there where you can get a sandwich for free and there is actually a doctor what will sit and listen to you and not just give you a prescription and rush you out the door” ’*

(PEHP, 49 years, England)

While specialist primary care centres for PEH often have longer appointment times than mainstream GP services, the participants really appreciated the flexibility the outreach settings afforded GPs to give time to listen to their concerns and address their needs.

#### Breaking down barriers between GPs and PEH

The organisational culture of the outreach settings broke down barriers between the GP and the participants in several ways: GPs were in a regular office space, not a clinical room, GPs sat with the participants in the social spaces and were able to talk informally with people as they ate food, drank tea, and used the computers, encouraging them to come to see the GP; and GPs were indistinguishable from the staff/volunteers in appearance, so the participants found them much more accessible than in the formality of a normal GP practice:

*‘The GPs are just like us — they sit out here* [in the social space] *and just chat to people until they trust them and then they will go and see the GP.’*

(V, Scotland)

*‘It (GP outreach) is far more approachable, you know, there doesn’t seem to be … I-ama-doctor, I-am-a-nurse. You know, it’s I-ama-person, you-are-a-person, what’s wrong with you? Which is the way it should be.’*

(PEHP, 58 years, Scotland)

*‘Here* [the outreach setting] *it is far more approachable and friendly sort of atmosphere … a bit less of a white coat syndrome.’*

(PEHP, 46 years, Scotland)

## DISCUSSION

### Summary

This study examined the experiences of PEH in accessing GP services in community outreach settings in Scotland and England, including staff/volunteers’ views on the strengths and weaknesses of GP outreach services. The study found that the organisational environment is important in enabling PEH to engage with GP services, challenging the notion that lack of engagement with primary care services by PEH is a result of people having ‘chaotic’ lives.^19^ The physical built space and the organisational environment in the outreach setting were the most important factors in enabling PEH to engage with GP outreach services. Combined, these two factors created a space where professional barriers between the GP and patients were flattened, and time was made available to nurture a therapeutic relationship. In addition, PEH were supported by staff/volunteers in the social areas to engage with the GP. While this study did not examine clinical outcomes for PEH, it can be assumed that engagement with GP services is a first step to accessing preventive care and treatment.

The findings of this study have important implications for clinical practice, as they call attention to the importance of the physical and organisational environment of healthcare delivery beyond merely the communication skills of the individual GP. While many studies have demonstrated the ways that inflexible systems and stigma act as a barrier to primary health care for PEH,^15^ this study highlights the importance of the built and social environment. In order for PEH to be able to engage with primary healthcare services, the waiting room space needs to have flexible time scales, meaningful activity available, staff/volunteer support, and a welcoming atmosphere. It is also advantageous if services and facilities are together in one place, as PEH do not prioritise healthcare needs over other immediate needs.^20^^,^^21^

### Strengths and limitations

In exploring the perspectives of PEH and staff/volunteers in community outreach settings, three different settings were used in Scotland and England, which enabled comparison within different practice contexts. Saturation of in-depth data occurred after 20 interviews and two focus groups, with no new themes emerging. The use of rigorous framework analysis increased the trustworthiness of the data.

A purposeful sample would have ensured that a wider range of different experiences were heard but it was only possible to recruit from a convenience sample given the difficulty with recruiting PEH in a community context. This study cannot provide comment on the literature around stigma, since the GPs who led the community outreach services all had a specialist interest in homelessness and it can be assumed that they held positive attitudes towards patients with complex needs.

### Comparison with existing literature

Previous research has found that PEH do not access mainstream GP services because of the inflexibility of the healthcare system and difficulties in building a relationship with GPs.^22^^,^^23^ In particular, the rigidity of the appointment system and the complexity of health and homelessness services make it difficult for PEH to navigate systems and engage meaningfully with preventive care and treatment options. While specialist primary care centres for PEH offer more flexibility, many PEH do not access any primary health care, waiting until they require emergency hospital admission.^24^ Health standards for commissioners/service providers recommend that primary care is central to improving the health care of PEH through vaccination, prevention, and screening for chronic conditions.^25^ This requires improved advocacy, interprofessional working, and engagement with the wider healthcare system beyond immediate medical treatment.^26^ This can only happen if PEH and other marginalised groups are able to engage with primary care services in meaningful ways.^5^ Relationships are seen as key to improving care,^22^ and education in trauma-informed care is increasingly seen as a positive way of educating staff about how to build better relationships with PEH. Another suggestion recommended by the health standards^25^ is that GP receptionists could become patient champions, ensuring access to primary care services. While these are positive steps forward, findings from the current study suggest that therapeutic relationships do not develop in a vacuum but are shaped by the physical and organisational environment of the service. As VanHeuvelen states,^27^ ‘the physical environment operates as a dynamic force that influences organisational members’ local cultural practices’. In order for local cultural practices and relationship dynamics between GPs and PEH to change, attention needs to focus on the local organisational environment.

One theoretical approach that has been shown to improve engagement with health services for PEH is the notion of psychologically informed environments. This approach calls attention to therapeutic relationships, staff training, the social and physical environment, psychological frameworks, and evidence generating practice,^28^ but further work is needed to more fully understand psychologically informed environments in a primary healthcare context. It should also be noted that just altering the physical structure of an organisation does not equate to altering the rules, culture, or policies of that organisation.^27^

### Implications for research and practice

This study reveals the importance of the waiting room environment when engaging PEH. Participants valued the opportunity for meaningful activity while waiting for the GP. Anxiety-provoking environments made them less likely to engage with health care. Although mainstream and specialist primary care centres for PEH will not have the same opportunities as outreach settings, for example offering food, they can use these findings to help inform and plan the environment in their waiting space. A focus on a welcoming feel with support/volunteer worker presence is recommended.

Participants found it convenient to have many services, including the GP, all in one place. This promoted engagement with the GP. These findings are useful when designing services for PEH where particular emphasis should be placed on working together with housing, social work, and third sector colleagues to create a ‘one-stop shop’ model of care.

Participants valued flexibility at outreach settings, not only in appointments but also the availability of the GP to adapt their time and location to the individual patient’s needs. Services should aim to be flexible on an individual and organisational level to better meet the needs of PEH.

Lastly, this study has revealed the extent to which the environment can influence the relationship between GPs and PEH. Participants described a more fulfilling relationship with the GP when the consultation took place in a relaxed environment outside of the perceived ‘institutional’ norms. GPs caring for PEH should seek to work with them to build an environment that supports the development of stronger doctor–patient relationships within the confines of their current system.

Evaluation of a range of different GP outreach approaches, including the impact on the health outcomes of PEH, are recommended for future studies.
